# Hybrid Newton–Successive Substitution Method for Multiphase Rachford-Rice Equations

**DOI:** 10.3390/e20060452

**Published:** 2018-06-09

**Authors:** Ran Gao, Xiaolong Yin, Zhiping Li

**Affiliations:** 1 School of Energy Resource, China University of Geosciences, Beijing 100083, China; 2 Petroleum Engineering, Colorado School of Mines, Golden, CO 80401, USA

**Keywords:** multiphase equilibrium, Rachford-Rice equation, three-phase flash calculation, successive substitution, Newton method

## Abstract

In multiphase (≥3) equilibrium calculations, when the Newton method is used to solve the material balance (Rachford-Rice) equations, poorly conditioned Jacobian can lead to false convergence. We present a robust successive substitution method that solves the multiphase Rachford-Rice equations sequentially using the method of bi-section while considering the monotonicity of the equations and the locations of singular hyperplanes. Although this method is slower than Newton solution, as it does not rely on Jacobians that can become poorly conditioned, it can be inserted into Newton iterations upon the detection of a poorly conditioned Jacobian. Testing shows that embedded successive substitution steps effectively improved the robustness. The benefit of the Newton method in the speed of convergence is maintained.

## 1. Introduction

Petroleum reservoir fluids are multi-component mixtures primarily made of hydrocarbons, held subsurface at high-temperature and high-pressure conditions [1,2]. Although under most situations the phase behavior of these fluids can be adequately described by vapor-liquid two-phase equilibria, multiphase phenomena (number of phases ≥3) have also been widely observed. Some of them, such as wax precipitation [3,4,5,6], hydrate formation [7,8,9,10], and asphaltene precipitation [11,12,13,14] are of significant importance to oil and gas production and transportation. In enhanced oil recovery, mixtures of reservoir oil and injected gas, such as CO_2_, can also exhibit complex phase behaviors at low pressures [15,16,17]. For these reasons, research on multiphase equilibrium calculations is still very active.

Multiphase (≥3) equilibrium calculation includes phase-stability [18] and phase-split [19] calculation steps. These two steps are usually carried out in series, except for the class of methods that was started by Gupta et al. [20], where they are carried out simultaneously. In the phase-split calculation step, mass balance equations and fugacity equations are solved. These two sets of equations can be solved sequentially using the method of successive substitution: the mass balance equations are first solved for a given set of equilibrium ratios; then, the equilibrium compositions of the phases that were obtained from the solution of the mass balance equations are used to update the equilibrium ratios through each phase’s equation of state or activity correlation [21,22,23]. These two sets of equations can also be solved simultaneously by the Newton method [24]. Although the Newton method converges more rapidly than the method of successive substitution, its accuracy is often influenced by the initial guess. For this reason, Michelson [19] recommended to begin phase-split calculations with successive substitutions and then switch to the Newton method. Successive substitution method and Newton method for phase-split calculations are abbreviated as SS-PS and NM-PS in this study.

This study focuses on the solution of the mass balance equations, the first step in SS-PS. Mass balance equations can be either solved by the Newton method [21,22,23,25,26] or by formulating the equations into a problem of minimization [27,28,29]. They are usually expressed in the Rachford-Rice form [30], which, for the case of two-phase equilibria, is easy to solve owing to its monotonicity and bounds of solutions explicitly indicated by the poles [31]. Following the abbreviations of SS-PS and NM-PS, we will abbreviate Newton method for the Rachford-Rice equations as NM-RR, and the minimization method for the Rachford-Rice equation as MM-RR. For multiphase (≥3) Rachford-Rice equations, the Newton method that was presented in Leibovici and Neoschil [26] is a widely-used NM-RR. Their method uses the locations of singular hyperplanes of the Rachford-Rice equations to confine the Newton iterations. The result is a robust convergence toward a unique solution in the multi-dimensional hypervolume, including the “negative flash” [32] region. It was found, however, by Okuno and coworkers [29], that Leibovici and Neoschil’s method would occasionally fail to converge to the correct answer, because the condition of the Jacobian becomes too large when the Newton steps are carried out very close to the singular hyperplanes. Okuno et al.’s method, which is based on the principle of minimization, uses the condition of non-negative phase compositions to further constrain the solutions and can overcome the problem of poorly conditioned Jacobians. Their method also converges with a fewer number of iterations as compared to the Leibovici and Neoschil’s method. The method of Okuno et al. is one example of the latest MM-RR.

In this study, we present a successive-substitution method to solve the multiphase Rachford-Rice equations that we will mention henceforth as SS-RR. Considerations of monotonicity and the locations of singular hyperplanes help this method to achieve robust convergence. The speed of convergence is slow when compared to NM-RR. However, as it does not use Jacobian or any derivatives, it can achieve convergence where NM-RR of Leibovici and Neoschil [26] cannot, due to poorly conditioned Jacobians. A hybrid code that combines the benefits of SS-RR and NM-RR is also presented. Section 2 is a presentation of the SS-RR method. In Section 3, we present the hybrid Newton-successive substitution method (NSS-RR) and two examples for which NM-RR could not converge to the correct answers, whereas both SS-RR and NSS-RR did. In Section 4, we present comparisons to show that the results from our calculations agree with those that were reported in the literature, including a complete phase-split calculation for an oil-gas-water system with equation of state and Henry’s law.

## 2. Successive Substitution Method for Multiphase Rachford-Rice Equations

In this study, we use ${\tilde{n}}^{j}$ to denote the mole fraction of phase *j* in a multiphase, multicomponent mixture, $x_{i}^{j}$ to denote the mole fraction of component *i* in phase *j*, and $z_{i}$ to denote the mole fraction of component *i* in the mixture. $N_{C}$ is the total number of components and $N_{P}$ is the total number of phases. The equilibrium ratio of component *i* between phase α and phase β is defined as $K_{i}^{\alpha \beta }=x_{i}^{\alpha }/x_{i}^{\beta }$. The multiphase Rachford-Rice equations that are derived from mass balances are

(1) $$\begin{array}{cc} \sum_{i=1}^{N_{C}}\frac{(1-K_{i}^{j1})z_{i}}{1-\sum_{k=2}^{N_{P}}{\tilde{n}}^{k}\left(1-K_{i}^{k1}\right)}=0 & j=2,\;\cdots ,\;N_{P} \end{array}$$

Here, selection of reference phase “1” is arbitrary, but it should contain every component of the system. While the selection of reference phase does not affect the results, it does affect convergence path and speed. We recommend to select reference phase to avoid extreme values of equilibrium ratios.

For a given set of equilibrium ratios $K_{i}^{j1}$ ($i=1,\;2,\;\cdots ,\;N_{C}$, $j=2,\;\cdots ,\;N_{P}$) and mole fractions $z_{i}$, Equation (1) is solved to give ${\tilde{n}}^{2}$ through ${\tilde{n}}^{N_{P}}$. Then, the compositions of phases are obtained using

(2) $$\begin{array}{ccc} x_{i}^{j}=\frac{z_{i}K_{i}^{j1}}{1+\sum_{k=2}^{N_{P}}{\tilde{n}}^{k}\left(K_{i}^{k1}-1\right)} & i=1,\;2,\;\cdots ,\;N_{C} & j=1,\;2,\;\cdots ,\;N_{P} \end{array}$$

In this study, we introduce

(3) $$\begin{array}{cc} \xi_{i}^{j}=K_{i}^{j1}-1 & j=2,\;\cdots ,\;N_{P} \end{array}$$

to simply Equation (1) into a set of fractional equations

(4) $$F_{j}\left({\tilde{n}}^{2},\cdots ,{\tilde{n}}^{N_{P}}\right)=\begin{array}{cc} \sum_{i=1}^{N_{C}}\frac{\xi_{i}^{j}z_{i}}{1+\sum_{k=2}^{N_{P}}\xi_{i}^{k}{\tilde{n}}^{k}}=0 & j=2,\;\cdots ,\;N_{P} \end{array}$$

The values of $\xi_{i}^{j}$ vary between −1 and ∞.

Although Equation (4) appears simple, formulating a robust numerical solution is rather complex, because of the presence of $N_{C}$ hyperplanes

(5) $$\begin{array}{cc} 1+\sum_{k=2}^{N_{P}}\xi_{i}^{k}{\tilde{n}}^{k}=0 & i=1,\;2,\;\cdots ,\;N_{C} \end{array}$$

on which Equation (4) becomes singular. Precarious application of the Newton method, without proper consideration of the hyperplanes, can lead to non-convergence or convergence toward non-physical solutions. Leibovici and Neoschil [26] recognized that the physical constraint on the mole fractions of the phases, $0\leq {\tilde{n}}^{j}\leq 1$ and $\sum_{j=2}^{N}{\tilde{n}}^{j}<1$, defines a hypertetrahedron in the $\left[{\tilde{n}}^{2},\;\ldots ,\;{\tilde{n}}^{N_{P}}\right]$ space, and this hypertetrahedron cannot be dissected by any of the hyperplanes. This property ensures that $F_{j}$ in Equation (4) are continuously differentiable in the hypervolume

(6) $$1+\xi_{i}^{j}{\tilde{n}}^{j}>0$$

that encloses the hypertetrahedron of the physically admissible solutions and the immediately adjacent negative flash region. By starting the initial guess within the hypervolume defined by Equation (6) and relaxing the Newton steps such that they do not cross the singular hyperplanes, Leibovici and Neoschil [26] were able to ensure that their Newton method does not generate a solution outside of Equation (6). However, the condition of $F_{j}$ being continuously differentiable does not guarantee that the Jacobian that is needed by the Newton method is always well conditioned. When Newton iteration approaches the boundaries of Equation (6), the Jacobian can become nearly singular, causing the Newton method to fail [29].

The abovementioned limitation of the Newton method motivated us to seek a method that does not require the computation of Jacobian or any derivatives of $F_{j}$. Our method began with the recognition that since

(7) $$\frac{\partial F_{j}}{\partial {\tilde{n}}^{j}}=\sum_{i=1}^{N_{C}}-\frac{{\left(\xi_{i}^{j}\right)}^{2}z_{i}}{{\left[1+\sum_{k=2}^{N_{P}}\xi_{i}^{k}{\tilde{n}}^{k}\right]}^{2}}<0$$

**Figure 1:**
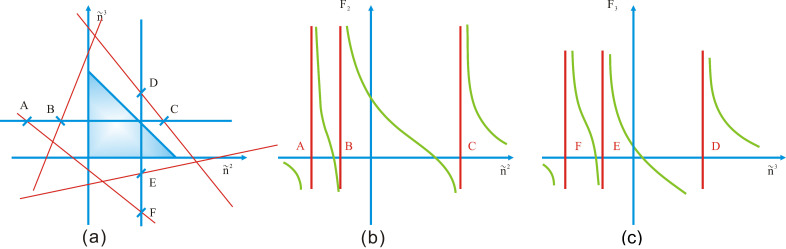
(**a**) The blue triangle is the domain of physically admissible solutions, surrounded by four singular lines. The variation in $F_{2}$ along the horizontal dashed line A-B-C and that in $F_{3}$ along the vertical dashed line D-E-F are shown in (**b**,**c**), respectively. Both $F_{2}$ and $F_{3}$ decrease monotonically with increasing ${\tilde{n}}^{2}$ and ${\tilde{n}}^{3}$. The monotonic trends are separated by poles at A, B, C and D, E, F.

$F_{j}$ must be monotonically decreasing in the direction of increasing ${\tilde{n}}^{j}$. Because of this property, when ${\tilde{n}}^{j}$ is increased to approach a singular hyperplane while all the other variables are kept constant, $F_{j}\to -\infty$. When ${\tilde{n}}^{j}$ is decreased to approach a singular hyperplane while all the other variables are kept constant, $F_{j}\to +\infty$. Additionally, when ${\tilde{n}}^{j}\to +\infty$ while all other variables are kept finite, $F_{j}$ decreases and approaches zero; when ${\tilde{n}}^{j}\to -\infty$ while all other variables are kept finite, $F_{j}$ increases and approaches zero. Figure 1 shows, qualitatively for a three-phase system, the monotonicity of $F_{2}$ and $F_{3}$ to ${\tilde{n}}^{2}$ and ${\tilde{n}}^{3}$.

The monotonicity of $F_{j}$ to ${\tilde{n}}^{j}$, but not the other variables motivated us to design a successive-substitution method to solve Equation (4), as follows. First, an initial guess is made within the hypertetrahedron of physically admissible solutions, ${\tilde{n}}_{0}^{2}={\tilde{n}}_{0}^{3}=\;\cdots \;={\tilde{n}}_{0}^{N_{P}}=1/N_{P}$. Here, the subscripts to ${\tilde{n}}^{j}$ denote the number of iterative substitutions. We begin the first iteration by holding the values of ${\tilde{n}}_{0}^{3}$ through ${\tilde{n}}_{0}^{N_{P}}$ constant and seek ${\tilde{n}}_{1}^{2}$ to satisfy $F_{2}\left({\tilde{n}}_{1}^{2},{\tilde{n}}_{0}^{3},\;\cdots ,{\tilde{n}}_{0}^{N_{P}}\right)=0$. Once ${\tilde{n}}_{1}^{2}$ is identified, we hold ${\tilde{n}}_{1}^{2}$ and ${\tilde{n}}_{0}^{4}$ through ${\tilde{n}}_{0}^{N_{P}}$ constant and seek ${\tilde{n}}_{1}^{3}$ to satisfy $F_{3}\left({\tilde{n}}_{1}^{2},{\tilde{n}}_{1}^{3},{\tilde{n}}_{0}^{4},\;\cdots ,{\tilde{n}}_{0}^{N_{P}}\right)=0$. This process continues till $F_{N_{P}}=0$ is solved and subscripts of all ${\tilde{n}}^{j}$ are updated to level “1”, which finishes the first iteration. The second iteration begins by holding the values of ${\tilde{n}}_{1}^{3}$ through ${\tilde{n}}_{1}^{N_{P}}$ constant and seek ${\tilde{n}}_{2}^{2}$ to satisfy $F_{2}\left({\tilde{n}}_{2}^{2},{\tilde{n}}_{1}^{3},\;\cdots ,{\tilde{n}}_{1}^{N_{P}}\right)=0$, and ends when all the subscripts of ${\tilde{n}}^{j}$ are updated to level “2”. Iteration would then begin at level “3”, and continue till all ${\tilde{n}}^{j}$ are converged.

With this successive-substitution strategy, solution of Equation (4) is reduced to successive solutions of single-variable equation $F_{j}\left({\tilde{n}}^{j}\right)=0$. Recognizing that $F_{j}\left({\tilde{n}}^{j}\right)$ is monotonic between its poles, we used the following method to solve $F_{j}\left({\tilde{n}}^{j}\right)=0$:

- Evaluate $F_{j}\left({\tilde{n}}_{m}^{2},\;\cdots ,\;{\tilde{n}}_{m}^{j-1},\;\left[{\tilde{n}}_{m-1}^{j*}\right],\;{\tilde{n}}_{m-1}^{j+1},\;\cdots ,\;{\tilde{n}}_{m-1}^{N_{P}}\right)$. Here, *m* refers to the level of iteration and ${\tilde{n}}^{j^{*}}$ in the square bracket is the variable that needs to be updated to level “*m*” such that $F_{j}\left({\tilde{n}}_{m}^{2},\;\cdots ,\;{\tilde{n}}_{m}^{j-1},\;{\tilde{n}}_{m}^{j},\;{\tilde{n}}_{m-1}^{j+1},\;\cdots ,\;{\tilde{n}}_{m-1}^{N_{P}}\right)=0$.
- Determine the direction along which to vary ${\tilde{n}}^{j^{*}}$. If $F_{j}\left({\tilde{n}}_{m}^{2},\;\cdots ,\;{\tilde{n}}_{m}^{j-1},\;\left[{\tilde{n}}_{m-1}^{j^{*}}\right],\;{\tilde{n}}_{m-1}^{j+1},\;\cdots ,\;{\tilde{n}}_{m-1}^{N_{P}}\right)>0$, ${\tilde{n}}^{j^{*}}$ should be increased. If, however, $F_{j}\left({\tilde{n}}_{m}^{2},\;\cdots ,\;{\tilde{n}}_{m}^{j-1},\;\left[{\tilde{n}}_{m-1}^{j*}\right],\;{\tilde{n}}_{m-1}^{j+1},\;\cdots ,\;{\tilde{n}}_{m-1}^{N_{P}}\right)<0$, ${\tilde{n}}^{j^{*}}$ should be decreased.
- Check whether a solution exists along the direction of increasing (or decreasing) ${\tilde{n}}^{j^{*}}$. This is achieved by performing a line search to see whether increasing (or decreasing) ${\tilde{n}}^{j^{*}}$ would lead to an intersection with a singular hyperplane. If ${\tilde{n}}^{j^{*}}$ needs to be increased and increasing ${\tilde{n}}^{j^{*}}$ generates intersections with singular hyperplanes, one solution must exist between the current ${\tilde{n}}^{j^{*}}$ and the nearest intersection, because, as ${\tilde{n}}^{j^{*}}$ approaches the intersection, $F_{j}\left({\tilde{n}}^{j*}\right)$ approaches −∞. If ${\tilde{n}}^{j^{*}}$ needs to be increased, but there is no intersection with singular hyperplanes along the direction of increasing ${\tilde{n}}^{j^{*}}$, there is no solution to $F_{j}\left({\tilde{n}}_{m}^{2},\;\cdots ,\;{\tilde{n}}_{m}^{j-1},\;{\tilde{n}}_{m}^{j},\;{\tilde{n}}_{m-1}^{j+1},\;\cdots ,\;{\tilde{n}}_{m-1}^{N_{P}}\right)=0$ and the calculation stops. Similar criteria apply when ${\tilde{n}}^{j^{*}}$ needs to be decreased.
- When solution exists, vary ${\tilde{n}}^{j^{*}}$ to find ${\tilde{n}}_{m}^{j^{*}}$. Because there is one and only one solution between ${\tilde{n}}_{m-1}^{j^{*}}$ and the nearest intersection with singular hyperplanes, a bi-section method is used. Testing begins at the midpoint between ${\tilde{n}}_{m-1}^{j^{*}}$ and the nearest intersection. The interval that contains the solution is continuously halved until $F_{j}$ evaluated at the midpoint of the interval becomes zero within a prescribed precision.

Successive substitution and bi-section methods that are presented above are very robust. As the initial guess is within the hypervolume defined by Equation (6) and solution search is bounded by the singular hyperplanes, this algorithm will never generate a solution outside the hypervolume. A flow chart for this algorithm is provided in Appendix B.

At the end of this section, we present an example that involves gas, oil, and water phases. The system that is considered here only consists of three components: methane, *n*-butane, and water. The pressure is 13.8 MPa and the temperature is 93.3 °C. Methane, *n*-butane, and moisture are present in the gas phase (g = 1); methane, *n*-butane, and dissolved water are present in the oil phase (o = 2); and, dissolved methane, dissolved *n*-butane, and water are present in the water phase (w = 3). The equilibrium ratios needed by the calculation were obtained from solubility data of pure hydrocarbons in pure water [33], published equilibrium ratios for hydrocarbons in a retrograde gas [34], moisture content in natural gas [35], and water solubility data in hydrocarbons [36]. At the pressure and temperature of interest, the mole fractions of methane and *n*-butane in the water phase are: $x_{C_{1}}^{w}=1.6\times 10^{-3}$, $x_{nC_{4}}^{w}=1.5\times 10^{-4}$. Assuming that the equilibrium ratios for methane and *n*-butane, between gas and oil phases, are not affected by whether these phases contain water or not, we obtained $K_{C_{1}}^{go}=2.181$, $K_{nC_{4}}^{go}=0.350$. Data on moisture content in natural gas suggests that at the pressure and temperature of interest, the mole fraction of water in the gas phase should be $x_{H_{2}O}^{g}=2.894\times 10^{-2}$. Correlation in Hibbard and Schalla [36] suggests that mole fraction of water in the oil phase can be set to $x_{H_{2}O}^{o}=0.004$. The calculated compositions of the three phases and the equilibrium ratios are listed in Table 1, together with their corresponding $\xi_{i}^{j}$.

This example was solved using a mixture composition of $z_{C_{1}}=0.6$, $z_{nC_{4}}=0.35$ and $z_{H_{2}O}=0.05$. The initial guess was $\left({\tilde{n}}^{g},\;{\tilde{n}}^{o},\;{\tilde{n}}^{w}\right)=\left(1/3,\;1/3,\;1/3\right)$. The case converged within five iterations with convergence criterion $\left\|\left[\Delta {\tilde{n}}^{o},\Delta {\tilde{n}}^{w}\right]\right\|\leq 10^{-7}$.

**Figure 2:**
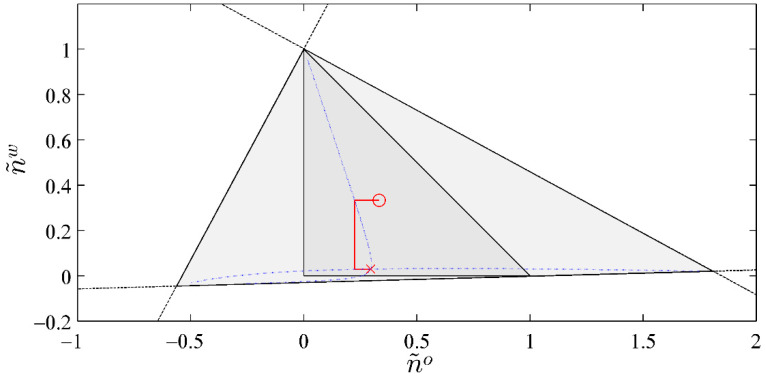
Red line ended with circle and cross marks the path of convergence for the three-component, three-phase example presented in Table 1. Circle is the initial guess and cross is the converged solution. The smaller shaded triangle is the domain of the physically permissible solutions. The larger shaded triangle is the domain defined by Equation (6) including the negative flash region. The dashed lines are the singular lines. The dotted lines inside the larger shaded triangle correspond to $F_{2}=0$ and $F_{3}=0$, the intersection of which is the converged solution.

Figure 2 shows the path of convergence from the initial guess. Solution for ${\tilde{n}}^{g}$, ${\tilde{n}}^{o}$, and ${\tilde{n}}^{w}$ is (0.6725, 0.2981, 0.0294).

We note that this SS-RR method can be used to obtain, for three-phase equilibrium calculations, the lines on which $F_{2}=0$ and $F_{3}=0$. These lines are of special interest, because their intersection is the needed solution. Such lines have been used by Haugen et al. [37] and Li and Firoozabadi [24] in order to explain their area-based bi-section method to solve $F_{2}$ and $F_{3}$. The dotted lines in Figure 2 mark the locations where $F_{2}=0$ and $F_{3}=0$, respectively. They were constructed from the paths of convergence of 1780 SS-RR calculations, each with a different initial guess within the area that is defined by Equation (6). These calculations all converged to the same solution, which indicates that initial guess is not important as long as it is bounded by Equation (6).

## 3. Hybrid Newton–Successive Substitution Method

The convergence speed of the above SS-RR method is slow when compared to the NM-RR method of Leibovici and Neochil [26]. However, as SS-RR is robust and it does not require the Jacobian or any derivative of $F_{j}$, it can be integrated into NM-RR to handle situations with poorly conditioned Jacobians.

In hybrid Newton-Successive substitution (NSS-RR) method, the Newton step is identical to that in Leibovici and Neochil [26]. Prior to each Newton step, however, the condition number of the Jacobian matrix, κ, is evaluated. A Newton iteration is carried out if κ is less than a prescribed value and a successive-substitution iteration is carried out if otherwise. A flow chart for NSS-RR is provided in Appendix B.

When starting a success-substitution iteration, the result from the previous Newton step can be directly used. When starting a Newton step after a successive-substitution iteration, however, care should be taken because at the end of successive substitution $F_{N_{P}}=0$. Assume that $\left[{\tilde{n}}_{m-1}^{2},\;\cdots ,\;{\tilde{n}}_{m-1}^{N_{P}}\right]$ is the result of the m−1-th iteration and $\left[{\tilde{n}}_{m}^{2},\;\cdots ,\;{\tilde{n}}_{m}^{N_{P}}\right]$ is the result of the m-th iteration, which is a successive-substitution. If the m+1-th iteration is a Newton step, we recommend not to use $\left[{\tilde{n}}_{m}^{2},\;\cdots ,\;{\tilde{n}}_{m}^{N_{P}}\right]$ to start the Newton step, but a combination of $\left[{\tilde{n}}_{m-1}^{2},\;\cdots ,\;{\tilde{n}}_{m-1}^{N_{P}}\right]$ and $\left[{\tilde{n}}_{m}^{2},\;\cdots ,\;{\tilde{n}}_{m}^{N_{P}}\right]$. We specifically used

(8) $${\tilde{n}}^{i^{*}}=\frac{i-1}{N_{P}-1}{\tilde{n}}_{m-1}^{i}+\frac{N_{P}-i}{N_{P}-1}{\tilde{n}}_{m}^{i}$$

to start the Newton step after successive-substitution. Geometrically, ${\tilde{n}}^{i^{*}}$ is the average of the points along the path of successive-substitution from $\left[{\tilde{n}}_{m}^{2},\;{\tilde{n}}_{m-1}^{3},\;\cdots ,\;{\tilde{n}}_{m-1}^{N_{P}}\right]$ to $\left[{\tilde{n}}_{m}^{2},{\tilde{n}}_{m}^{3},\cdots ,{\tilde{n}}_{m}^{N_{P}}\right]$. As each of these points makes one and only one $F_{j}$ zero, ${\tilde{n}}^{j^{*}}$ calculated from Equation (8) is guaranteed to separate from surfaces $F_{j}=0$, thus making it a good choice in our opinion to start the Newton step.

In what follows, we present two examples for which NM-RR of Leibovici and Neoschil [26] did not converge to the correct answers, whereas both SS-RR and NSS-RR were successful. The first example is a fifteen-component, three-phase mixture. The second example is a twenty-component, five-phase mixture. In NM-RR and NSS-RR, the relaxation parameter that regulates the Newton step when it intersects with the singular hyperplanes was set to 0.5. The maximum condition number in NSS-RR was set to 10^10^. In all of the methods, the convergence criterion set on the difference between vectors ${\tilde{n}}_{m-1}^{i}$ and ${\tilde{n}}_{m}^{i}$ was 10^−7^. Note that if the convergence criterion is tightened, then NM-RR can find the correct solutions for these examples. We are only using these examples to compare NM-RR and NSS-RR under the same convergence criterion.

**Figure 3:**
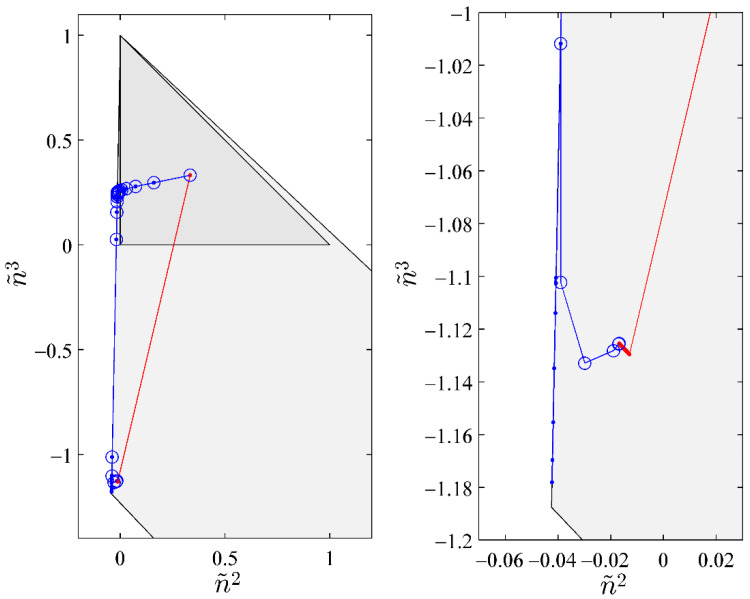
Paths of convergence for the case presented in Table 2. Red line with dots is the path of SS-RR, blue line with dots is the path of NM-RR, and blue line with circles is the path of the hybrid method. The graph on the left shows the entirety of the paths and the graph on the right focuses on the final convergence.

**Figure 4:**
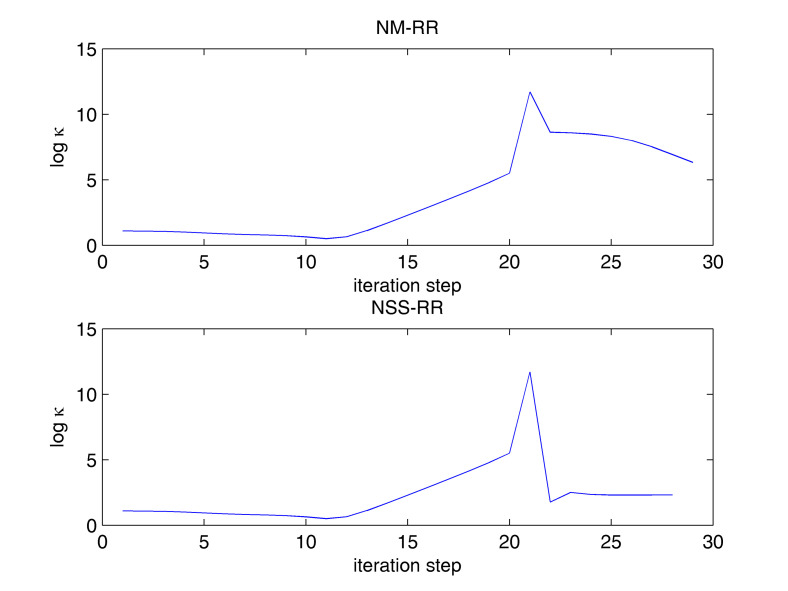
Variations in the condition number of Jacobian during NM-RR and NSS-RR iterations for the fifteen-component, three-phase example.

Table 2 gives the composition of the fifteen-component, three-phase mixture, the equilibrium ratios, and the equilibrium composition of the three phases. For this example, both NSS-RR and SS-RR converged to the correct root (−0.01686263294, −1.1254155641). NM-RR of Leibovici and Neoschil [26] however converged to a wrong root (−0.04078420653, −1.1004615900). As shown in Figure 3, many Newton steps were carried out very close to a singular line of the mixture. At the 21st step, a very large condition number of 5.0977 × 10^11^ was encountered. The hybrid method carried out a successive-substitution step at this location and the subsequent Newton steps converged to the correct solution. The NM-RR method, on the other hand, lost accuracy at this location and it converged to a wrong solution. Figure 4 shows the variations in the condition number of the Jacobian during iterations. NM-RR finished in 29 iteration steps and NSS-RR finished in 28 iteration steps. The 21st step in NSS-RR is the only successive-substitution step carried out.

**Figure 5:**
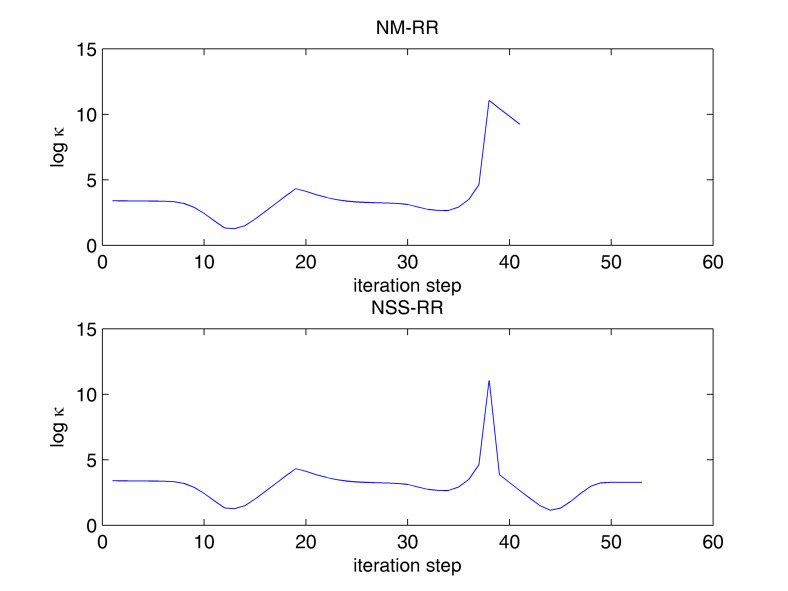
Variations in the condition number of Jacobian during NM-RR and NSS-RR iterations for the twenty-component, five-phase example.

Table 3 gives the composition of the twenty-component, five-phase mixture, the equilibrium ratios, and the equilibrium compositions of the five phases. For this example, both NSS-RR and SS-RR converged to the correct root (−0.00538660799, −0.00373696250, −0.00496311432, −0.00415370309). NM-RR of Leibovici and Neoschil [26], however, converged to a wrong root (−0.00287415017, −0.00392609623, −0.00798417906, −0.00350187286). Figure 5 shows that, at the 38th step, the condition number of the Jacobian reached 3.52 × 10^11^. Upon detecting this large condition number, NSS-RR performed a single successive-substitution step, which helped the following Newton steps to converge to the correct solution using a total of 54 iterations. NM-RR of Leibovici and Neoschil [26], however, lost accuracy after the 38th step and converged to a wrong solution.

As observed from the above examples, as SS-RR does not use any Jacobians nor derivatives, it is a useful method to switch to upon detection of a poorly conditioned Jacobian. In our hybrid NSS-RR, successive substitution is only activated on rare occasions, and hence it does not add significantly to the computational time. Checking the condition numbers of Jacobians, on the other hand, generated a non-negligible overhead to the algorithm. In average, the run time of NSS-RR is about 1.4 times of that of NM-RR.

## 4. Validations and A Three-Phase Equilibrium Calculation

After proving the robustness of the developed SS-RR and NSS-RR, in this section, we first compare our solutions of three-phase Rachford-Rice equations to those that are available in the literature. Then, we combine the developed solver with equation of state and Henry’s law to solve a complete oil-gas-water three-phase equilibrium.

### 4.1. Validations of Three-Phase Solutions

Three cases of three-phase equilibria from Nichita et al. [38] are used here to validate our solutions of three-phase Rachford-Rice equations. The first case is a ternary mixture containing CO_2_, CH_4_, and normal-hexadecane (nC_16_). The second case is a sour gas system with six components, the details of which can be found in Robinson et al. [39]. The third case is a quaternary mixture, the phase behavior of which was studied by Kohse and Heidemann [40]. Table 4, Table 5 and Table 6 list the compositions of the mixtures and the equilibrium compositions of the three phases. 

Equilibrium ratios that were obtained from these tables were passed to our multiphase Rachford-Rice equation solvers. We note here that all solvers, NM-RR, SS-RR, and hybrid NSS-RR, converged on these cases and gave identical results. In Table 7, we compare our results to those that were reported in Nichita et al. [38]. This comparison shows that results from our algorithm are in very good agreement with previously reported solutions.

In a recent work by Okuno et al. [29], four three-phase examples were presented (Table 1 in reference [29]), including flash and negative flash near critical points. We note that our solvers can achieve convergence on all four examples. Although a quantitative comparison is not possible because the authors did not report the final mole fractions, our converged solutions do visually agree with that are those presented in the figures of reference [29].

### 4.2. Three-Phase Equilibrium of An Oil-Gas-Water System

In this section, we combine multiphase Rachford-Rice solvers with equation of state (EOS) and Henry’s law, and use the SS-PS approach to solve an oil-gas-water equilibrium. Water exists extensively in many hydrocarbon reservoirs. Water in reservoir may come from initial water traps, migration from nearby aquifers, or water injection for improved/enhanced oil recovery. The presence of water can affect the phase behavior of reservoir fluids, because some gases, in particular, CO_2_ and H_2_S, are soluble in the aqueous phase. Three-phase equilibrium calculations are needed to correctly describe the phase behaviors of such systems [41,42].

In this calculation, fugacities of components in the vapor and liquid phases were modeled using Peng-Robinson EOS following standard procedures. Fugacities of components in the water-rich phase were modeled using Henry’s law [43]. Henry’s law for a component sparingly soluble in the aqueous phase is

(9) $$f_{i}^{w}=x_{i}^{w}H_{i},i\neq w$$

where $H_{i}$ is Henry’s law constant of component *i* in the aqueous phase. Details of Henry’s law can be found in Appendix A. The fugacity of water in the aqueous phase can be obtained from the fugacity of the solutes using the Gibbs-Duhem equation, as in Prausnitz [44].

(10) $$f_{H_{2}O}^{w}=x_{H_{2}O}^{w}\Phi^{ws}p_{vp}^{ws}(\int_{p_{vp}^{ws}}^{p}\frac{v_{m}}{RT}dp)$$

$\Phi^{ws}$ is the fugacity coefficient of pure water at the saturation vapor pressure $p_{vp}^{ws}$ and $v_{m}$ is the molar volume of pure water. The fugacity coefficient of water $\Phi^{ws}$ is calculated by the Chou equation, reported by Rowe and Chou [45], as follows:

(11) $$\Phi^{ws}=\{\begin{array}{ll} 0.9958+9.68330\times 10^{-5}T^{\prime }-6.175\times 10^{-7}T^{\prime 2}-3.08333\times 10^{-10}T^{\prime 3} & T^{\prime }>90^{\circ }F\\ 1 & T^{\prime }<90^{\circ }F \end{array}$$

For a three-phase system with $n_{c}$ components, the following equations must be satisfied:

(12) $$f_{i}^{v}=f_{i}^{l}{\begin{array}{cc} & i=1,\;\cdots n \end{array}}_{c}$$

(13) $$f_{i}^{w}=f_{i}^{l}\begin{array}{cc} & i=1,\;\cdots n_{c} \end{array}$$

Li and Nghiem [46] defined the following sets of equilibrium ratios

(14) $$\begin{array}{cc} K_{i}^{vl}=\frac{x_{i}^{v}}{x_{i}^{l}} & i=1,\;\cdots N_{c} \end{array}$$

(15) $$\begin{array}{cc} K_{i}^{vw}=\frac{x_{i}^{v}}{x_{i}^{w}} & i=1,\;\cdots N_{c} \end{array}$$

that are consistent with our equilibrium ratios, as defined in Section 2. The three-phase Rachford-Rice equations for our vapor-liquid-water system, following Equation (1), are

(16) $$\sum_{i=1}^{N_{C}}\frac{(1-K_{i}^{vl})\cdot z_{i}}{1-(1-K_{i}^{vl})\cdot {\tilde{n}}^{v}-(1-K_{i}^{wl})\cdot {\tilde{n}}^{w}}=0$$

(17) $$\sum_{i=1}^{N_{C}}\frac{(1-K_{i}^{wl})\cdot z_{i}}{1-(1-K_{i}^{vl})\cdot {\tilde{n}}^{v}-(1-K_{i}^{wl})\cdot {\tilde{n}}^{w}}=0$$

The equilibrium calculation procedure began by passing initial guesses of equilibrium ratios to the Rachford-Rice equations. ${\tilde{n}}^{l}$ and ${\tilde{n}}^{w}$ solved from the Rachford-Rice equations were used to compute compositions of the phases: $x_{i}^{v}$, $x_{i}^{l}$, and $x_{i}^{w}$, following Equations (4) and (5). These compositions were then used to determine the fugacity coefficients $\Phi_{i}^{v}$ and $\Phi_{i}^{l}$ and the fugacity $f_{i}^{w}$, from which the equilibrium ratios were updated. The equilibrium ratios were again passed to the Rachford-Rice equations, until the results converge.

We applied the method that is presented above to a six-component mixture, the composition of which is shown in Table 8. Detailed description of this case can be referred to Li and Nghiem [46]. We conducted equilibrium calculation at 10 MPa and 100 °C. Our calculated results are in good agreement with results in the literature, as shown in Table 9.

To illustrate the difference between a three-phase equilibrium calculation and two-phase equilibrium calculations that discount the influence of the aqueous phase, we conducted a two-phase equilibrium calculation where water is removed from the mixture. The mole fractions of the non-aqueous components are reconstructed based on the relative fractions of them in the original mixture. The results of this two-phase calculation are also shown in Table 9. Comparison shows that although the two-phase approach neglecting the water component generated phase compositions that are similar to those from the three-phase calculation, the ratio between liquid and gas mole fractions, however, is noticeably higher. At equilibrium, the aqueous phase contains 14% of H_2_S and 2% of CO_2_ by mole, and is therefore highly corrosive. This corrosive nature can only be captured by a three-phase equilibrium calculation. 

## 5. Conclusions

This paper presents a successive-substitution method to solve multiphase Rachford-Rice equations. In this algorithm, Rachford-Rice equations are solved sequentially, and the solution of each individual Rachford-Rice equation is achieved using the method of bi-section. The direction of bi-section is determined by the monotonicity of the equation, whereas the limit of bi-section is determined by the locations of the poles. These considerations ensure that the algorithm always reliably converges to either a physically admissible solution or a solution in the adjacent negative flash region.

In compositional reservoir simulations, the number of phase equilibrium calculations performed is very large. Accuracy and robustness without a significant penalty on efficiency are therefore very important. A hybrid method was developed in order to combine the advantages of this successive-substitution method and the Newton method. In this hybrid method, a successive substitution step replaces a Newton step when the local Jacobian becomes poorly conditioned. This hybrid method can effectively suppress the errors in the Newton method of Leibovici and Neochil [26] owing to poorly conditioned Jacobian. In terms of computational time, successive substitution steps add very little to the overall computational cost. The computational time of this hybrid method is 1.4 times of that of the Newton method of Leibovici and Neochil [26], and this difference is mainly due to the overhead needed to check the condition numbers.

We presented seven examples to show the characteristics and the accuracy of the algorithm. The first example shows the convergence behavior of the successive-substitution method. The second and the third examples present the advantages of the hybrid method over the Newton method of Leibovici and Neochil [26]. In the rest of the examples, we verified our algorithm using results from the literature. Lastly, we combined the developed multiphase Rachford-Rice solvers with equations of states for the oil and gas phases and Henry’s law for the water phase and completed a three-phase flash calculation for an oil-gas-water system with sour gases. Our result again is in very good agreement with that previously reported. It shows specifically that a two-phase equilibrium calculation neglecting the dissolution of sour gases in the water phase is not a good approximation of a true three-phase flash calculation.

## Figures and Tables

**Table 1:** Compositions of phases and equilibrium ratios.

	$x_{i}^{g}$	$x_{i}^{o}$	$x_{i}^{w}$	$K_{i}^{go}$	$K_{i}^{gw}$	$\xi_{i}^{o}$	$\xi_{i}^{w}$
H_2_O	0.02894	0.004	0.99825	7.235	0.029	−0.862	33.494
CH_4_	0.74143	0.33995	0.0016	2.181	463.394	−0.541	−0.998
*n*-C_4_H_10_	0.22963	0.65605	0.00015	0.350	1530.867	1.857	−0.999

**Table 2:** Composition and equilibrium ratios of the 15-component, 3-phase example.

Component	$z_{i}$	$K_{i1}$	$K_{i2}$	$x_{i}^{1}$	$x_{i}^{2}$	$x_{i}^{3}$
1	0.0315583329803	1.8528741428663	1.8115659762243	0.4366767940810	0.8091071405424	0.7910688227638
2	0.4071270076623	0.2314055308386	0.6954909860157	0.3003165208873	0.0694949039355	0.2088674332287
3	0.4751941671726	0.5041709444335	0.0001084501767	0.2227137374279	0.1122857953373	0.0000241533442
4	0.0545811711566	0.0635482083897	0.0012603511720	0.0255077448600	0.0016209714859	0.0000321487161
5	0.0115700446895	0.4078908506272	0.0013474694481	0.0054220598442	0.0022116086020	0.0000073060600
6	0.0189113955704	0.5066231481075	0.0000038929319	0.0088630653086	0.0044902340485	0.0000000345033
7	0.0000455484805	27.1901689643580	0.0035219133166	0.0000271151486	0.0007372654706	0.0000000954972
8	0.0006404014374	0.0765095693423	0.0000171923836	0.0002991176091	0.0000228853595	0.0000000051425
9	0.0003675973687	0.1284992832837	0.0000021965300	0.0001717657315	0.0000220717734	0.0000000003773
10	0.0000037504895	1.4795557872248	0.0001633840436	0.0000017714844	0.0000026210100	0.0000000002894
11	0.0000002428846	12.7769884293417	0.0016090228536	0.0000001261729	0.0000016121100	0.0000000002030
12	0.0000001594408	13.7666844672269	0.0007523046170	0.0000000835080	0.0000011496277	0.0000000000628
13	0.0000000228589	52.4995561949013	0.0000798682401	0.0000000181866	0.0000009547879	0.0000000000015
14	0.0000000202543	33.9539240672109	0.0000023516037	0.0000000129031	0.0000004381100	0.0000000000000
15	0.0000001375537	5.1979194333821	0.0000127574489	0.0000000669487	0.0000003479939	0.0000000000009

**Table 3:** Composition and equilibrium ratios of the 20-component, 5-phase example.

Component	$z_{i}$	$K_{i1}$	$K_{i2}$	$K_{i3}$	$K_{i4}$	$x_{i}^{1}$	$x_{i}^{2}$	$x_{i}^{3}$	$x_{i}^{4}$	$x_{i}^{5}$
1	0.3817399509140	2.3788914318714	0.1826346252218	2.1341148433378	0.7101073236142	0.3851281921976	0.9161781565910	0.0703377430444	0.8219077915568	0.2734823498098
2	0.0764336433731	0.8354537404402	2.0684286685920	1.9043018392943	6.0440859895389	0.0786796419224	0.0657332011406	0.1627432269869	0.1498297868279	0.4755465214053
3	0.1391487737570	0.1155938461254	2.8473183476162	0.0144945209799	0.4369041160293	0.1384439969943	0.0160032740856	0.3941941327593	0.0020066794190	0.0604867521264
4	0.0643992218952	0.0062262830625	2.1383860381928	0.0442168936781	0.9918488866995	0.0640229919586	0.0003986252704	0.1369058721276	0.0028308978284	0.0635011332973
5	0.1486026004951	0.0022156584248	0.7946416111326	0.0787337170042	0.7768884555186	0.1468925975170	0.0003254638212	0.1167269703543	0.0115654002029	0.1141191632121
6	0.0417212486653	0.0115951444765	2.1603434367941	0.0560494950996	0.2134611795537	0.0413515667922	0.0004794773913	0.0893335859206	0.0023177344403	0.0088269542238
7	0.1227693500767	0.0064167472255	0.1593792034596	0.0770042412753	0.0239948965688	0.1207019216039	0.0007745137206	0.0192373761213	0.0092945598936	0.0028962301245
8	0.0213087870239	0.0038946321018	0.0335917624138	0.0025050231128	0.0218059421417	0.0209321985655	0.0000815232125	0.0007031494410	0.0000524356412	0.0004564463108
9	0.0016270350309	0.0134366496720	0.7223258415919	0.1031743167040	0.1708086119388	0.0016041800354	0.0000215548051	0.0011587406941	0.0001655101790	0.0002740077651
10	0.0021307432306	0.0008734024997	2.6132706480239	0.0022130957042	0.0932727495955	0.0021136824783	0.0000018460956	0.0055236243798	0.0000046777816	0.0001971489765
11	0.0000917810305	0.0108844870333	24.4065005309508	0.1928690729187	1.0014414881636	0.0000995608488	0.0000010836688	0.0024299319085	0.0000192022086	0.0000997043646
12	0.0000229831930	0.0305288385881	25.8494898790919	0.0588393075672	4.0996590670858	0.0000254194834	0.0000007760273	0.0006570806789	0.0000014956648	0.0001042112156
13	0.0000034782551	0.0184206758492	10.4748859551860	0.3556852336181	0.1045382819199	0.0000035608768	0.0000000655938	0.0000372997780	0.0000012665513	0.0000003722479
14	0.0000001126367	1.9556944123756	57.6425128090423	1.7486777932718	29.0578470200348	0.0000001699095	0.0000003322911	0.0000097940117	0.0000002971170	0.0000049372049
15	0.0000002344634	0.2874467036782	1.0419187660436	1.8885719459373	13.7002699311125	0.0000002477114	0.0000000712038	0.0000002580951	0.0000004678207	0.0000033937126
16	0.0000000038064	1.5356775373006	53.5513911183565	97.7361071036055	6.6483533942909	0.0000000128093	0.0000000196710	0.0000006859566	0.0000012519326	0.0000000851609
17	0.0000000173126	0.7574272230786	7.6910401287961	6.0072238022229	18.7742085574180	0.0000000197267	0.0000000149415	0.0000001517188	0.0000001185027	0.0000003703530
18	0.0000000281366	0.0074377713757	6.7681727478028	4.0574761982724	5.2779281096742	0.0000000295911	0.0000000002201	0.0000002002775	0.0000001200651	0.0000001561795
19	0.0000000042589	0.0004574024029	28.1394115659509	35.1553173521778	9.0540032759730	0.0000000060755	0.0000000000028	0.0000001709606	0.0000002135856	0.0000000550075
20	0.0000000024453	0.0847561330613	1.6486494033625	31.9676317062480	2.5158440811075	0.0000000029023	0.0000000002460	0.0000000047849	0.0000000927811	0.0000000073018

**Table 4:** Composition of CO_2_–CH_4_–nC_16_ ternary mixture [38] and equilibrium compositions of the three phases (*g*: gas; *l*_1_ and *l*_2_: liquids).

Component	$z_{i}$	$x_{i}^{g}$	$x_{i}^{l_{1}}$	$x_{i}^{l_{2}}$
C_1_	0.05	0.078112	0.036181	0.038707
nC_16_	0.05	0.000069	0.340224	0.004609
CO_2_	0.90	0.921819	0.623595	0.956683

**Table 5:** Composition of the sour gas system mixture [38,39], and equilibrium compositions of the three phases (*g*: gas; *l*_1_ and *l*_2_: liquids).

Component	$z_{i}$	$x_{i}^{g}$	$x_{i}^{l_{1}}$	$x_{i}^{l_{2}}$
C_1_	0.70592	0.726129	0.714973	0.065262
C_2_	0.06860	0.006352	0.072154	0.012393
C_3_	0.02967	0.000466	0.031333	0.003738
H_2_S	0.10559	0.008596	0.097494	0.897742
CO_2_	0.01996	0.004933	0.020621	0.019166
N_2_	0.07026	0.253524	0.063425	0.001699

**Table 6:** Composition of the quaternary mixture [38,40] and equilibrium compositions of the three phases (*g*: gas; *l*_1_ and *l*_2_: liquids).

Component	$z_{i}$	$x_{i}^{g}$	$x_{i}^{l_{1}}$	$x_{i}^{l_{2}}$
C_1_	0.50000	0.977599	0.308646	0.068425
nC_6_	0.02627	0.000001	0.227252	0.007764
H_2_S	0.41633	0.007900	0.363483	0.833419
CO_2_	0.05740	0.014499	0.100618	0.090391

**Table 7:** Mole fractions of the three phases (g: gas; *l*_1_ and *l*_2_: liquids) from this study and from Nichita et al. [38].

	The Ternary Mixture			The Sour Gas System			The Quaternary Mixture		
	This Study	[38]	Differences	This Study	[38]	Differences	This Study	[38]	Differences
${\tilde{n}}^{g}$	0.2908	0.2962	1.8%	0.0417	0.0407	2.5%	0.4484	0.4482	0.0%
${\tilde{n}}^{l_{1}}$	0.5700	0.5645	1.0%	0.9434	0.9447	0.1%	0.1009	0.1002	0.7%
${\tilde{n}}^{l_{2}}$	0.1392	0.1393	0.1%	0.0149	0.0146	2.1%	0.4507	0.4516	0.2%

**Table 8:** Composition of the six-component mixture.

Component	Composition (%)
C_1_	30
nC_5_	15
nC_10_	25
CO_2_	10
H_2_S H_2_O	10 10

**Table 9:** Results for the case with three phases and six components in presence of water.

	This Study			Li and Nghiem (1986)			Two-Phase	
	$x^{l}\%$	$x^{g}\%$	$x^{w}\%$	$x^{l}\%$	$x^{g}\%$	$x^{w}\%$	$x^{l}\%$	$x^{g}\%$
C_1_	22.868	65.174	0.001	22.884	65.220	0.001	23.286	66.365
nC_5_	21.676	4.936	0	20.216	4.603	0	20.585	4.687
nC_10_	35.919	0.663	0	35.889	0.662	0	35.959	0.664
CO_2_	9.145	16.873	0.020	9.148	16.879	0.021	9.239	17.047
H_2_S	10.906	11.220	0.141	10.906	11.219	0.141	10.929	11.243
H_2_O	0.958	1.416	99.773	0.958	1.417	99.839	/	/
	${\tilde{n}}^{l}$	${\tilde{n}}^{g}$	${\tilde{n}}^{w}$	${\tilde{n}}^{l}$	${\tilde{n}}^{g}$	${\tilde{n}}^{w}$	${\tilde{n}}^{l}$	${\tilde{n}}^{g}$
	69.20	21.75	9.05	69.26	21.70	9.04	77.45	22.55

**Figure A1:**
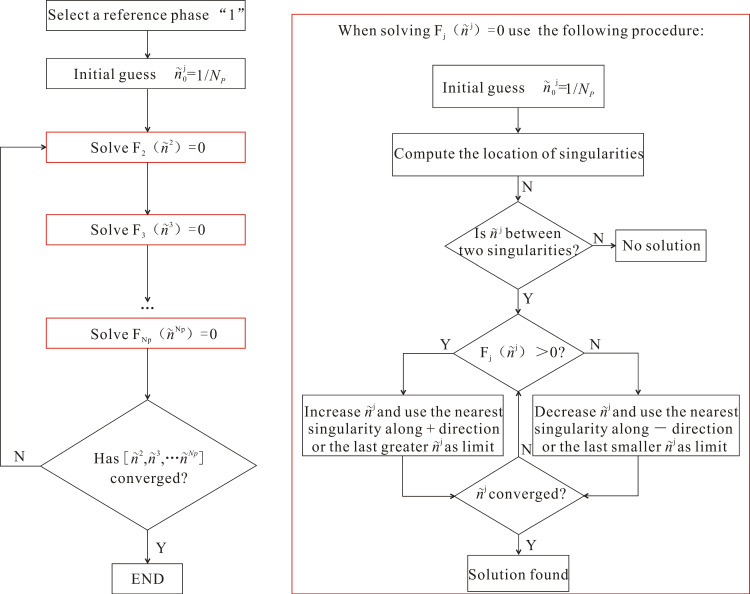
Flow chart of SS-RR.

**Figure A2:**
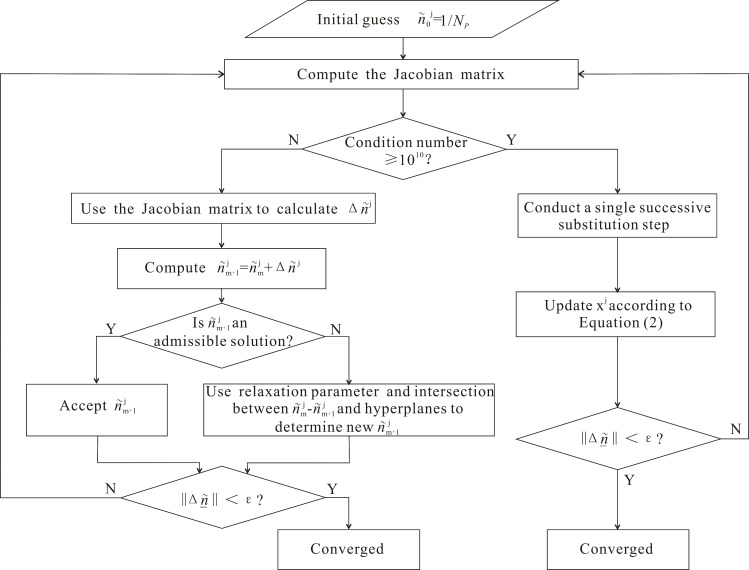
Flow chart of NSS-RR.

## Nomenclature

A(T)～H(T)	functions used to compute the specific volume v′
C	cohesive energy density of water
F	Rachford-Rice equations defined in Equation (4)
$f_{i}^{j}$	fugacity of component *i* in phase *j*
h	enthalpy departure
$H_{i}$	Henry’s law constant of component *i*
$K_{i}^{\alpha \beta }$	equilibrium ratio of component *i* between phase α and phase β
${\tilde{n}}^{j}$	mole fraction of phase *j*
$N_{c}$	number of components
$N_{P}$	total number of phases
p	pressure
R	universal gas constant
T	temperature
v′	specific volume
$v_{m}$	molar volume of pure water
$w_{NaCl}$	weight fraction of NaCl
$x_{i}^{j}$	mole fraction of component *i* in phase *j*
$z_{i}$	mole fraction of component *i* in the multiphase mixture
κ	condition number of Jacobian
$\xi_{i}^{j}$	defined in Equation (3) as $K_{i}^{j1}-1$
$\Phi_{i}$	fugacity coefficient of component *i*
*Superscripts*	
*g*	gas
*j*	phase index
*l*	liquid
*o*	oil
*s*	saturated
*w*	water
ws	water at saturation
α,β	phase index
*Subscripts*	
*c*	critical point
i,j,k	component index
*vp*	vapor pressure
*m*	level of iteration

## Appendix A. Henry’s Law

Henry’s law constant can be solved from a differential equation proposed by Smith and Van Ness [47]

(A1) $$d(\ln H_{i})=\frac{v_{mi}^{\infty }}{RT}dp+\frac{h_{i}^{v}-h_{i}^{\infty }}{RT^{2}}dT$$

In this equation, $v_{mi}^{\infty }$ is the partial molar volume of component *i* in the aqueous phase at infinite dilution, $h_{i}^{v}$ is the enthalpy of component *i* in the gas phase, and $h_{i}^{\infty }$ is the enthalpy of component *i* in the aqueous phase at infinite dilution. For a given temperature *T*, integration of Equation (A1) from $p^{0}$ to p gives

(A2) $$\ln H_{i}=\ln H_{i}^{0}+\frac{v_{mi}^{\infty }(p-p_{i}^{0})}{RT}$$

where $H_{i}^{0}$ is Henry’s law constant at the reference pressure $p_{i}^{0}$. Equation (A2) can also be written as

(A3) $$\ln H_{i}=\ln H_{i}^{*}+v_{mi}^{\infty }p/(RT)$$

where

(A4) $$\ln H_{i}^{*}=\ln H_{i}^{0}-v_{mi}^{\infty }p_{i}^{0}/(RT)$$

In this study, Equation (A3) was used to correlate Henry’s law constant from solubility data. $H_{i}^{*}$ is referred to as the reference Henry’s law constant. The molar volume at infinite dilution was calculated using the following correlation proposed by Lyckman et al. [48]

(A5) $$\frac{p_{ci}v_{mi}^{\infty }}{RT_{ci}}=0.095+2.35(\frac{Tp_{ci}}{CT_{ci}})$$

where *C* is the cohesive energy density of water given by:

(A6) $$C=\frac{\left(h^{w0}-h^{ws}+p_{vp}^{ws}v_{m}^{ws}-RT\right)}{v_{m}^{ws}}$$

$p_{vp}^{ws}$ is the saturated vapor pressure of water at temperature *T*, $v_{m}^{ws}$ is the molar volume of water at $p_{vp}^{ws}$ and *T*, and $h^{wo}-h^{ws}$ is the enthalpy departure of liquid water at $p_{vp}^{ws}$ and *T*. The saturated vapor pressure of water $p_{vp}^{ws}$ was calculated using the Buck equation [49]

(A7) $$p_{vp}^{ws}=0.61121\exp((18.678-\frac{T}{234.5})(\frac{T}{257.14+T}))$$

T is in ${}^{\circ }C$, and $p_{vp}^{ws}$ is in psi. Equation (A7) is applicable to liquid water: $T>0\;{}^{\circ }C$. When $T<0\;{}^{\circ }C$, for ice we used the following equation

(A8) $$p_{vp}^{ws}=0.61115\exp((23.036-\frac{T}{333.7})(\frac{T}{279.82+T}))$$

Enthalpy departure of liquid water $\left(h^{w0}-h^{ws}\right)$ were calculated from Yen-Alexander, reported in Poling et al. [50] in the form of:

(A9) $$h^{w0}-h^{ws}=T_{c}\frac{7-4.5688{\left(\ln(\frac{p_{vp}^{ws}}{217.6})\right)}^{0.333}}{1+0.004\ln(\frac{p_{vp}^{ws}}{217.6})}$$

The molar volume $v_{m}$ was estimated from the specific volume $v^{\prime }$, which, in turn, was from a correlation by Chou reported in Rowe and Chou [45].

(A10) $$\begin{array}{l} v^{\prime }=A(T)-p\cdot B(T)-p^{2}\cdot C(T)+w_{NaCl}\cdot D(T)+x^{2}\cdot E(T)-xp\cdot F(T)\\ -x^{2}p\cdot G(T)-\frac{1}{2}xp^{2}\cdot H(T) \end{array}$$

$v^{\prime }$ is the specific volume in ${\mathrm{cm}}^{3}/g$, T is the temperature in K, p is the absolute pressure in $\mathrm{kgf}/{\mathrm{cm}}^{2}$, and $w_{NaCl}$ is the weight fraction of NaCl in solution. The temperature functions are,

(A11) $$\begin{array}{l} A(T)=5.916365-0.01035794T+0.9270048\times 10^{-5}T^{2}\\ -1127.522/T+100674.1/T^{2} \end{array}$$

(A12) $$\begin{array}{l} B(T)=0.5204914\times 10^{-2}-0.10482101\times 10^{-4}T+0.8328532\times 10^{-8}T^{2}\\ -1.1702939/T+102.2783/T^{2} \end{array}$$

(A13) $$C(T)=0.118547\times 10^{-7}-0.6599143\times 10^{-10}T$$

(A14) $$D(T)=-2.5166+0.0111766T-0.170552\times 10^{-4}T^{2}$$

(A15) $$E(T)=2.84851-0.0154395T+0.223982\times 10^{-4}T^{2}$$

(A16) $$F(T)=-0.0014814+0.82969\times 10^{-5}T-0.12469\times 10^{-7}T^{2}$$

(A17) $$G(T)=0.0027141-0.15391\times 10^{-4}T+0.22655\times 10^{-7}T^{2}$$

(A18) $$H(T)=0.62158\times 10^{-5}-0.40075\times 10^{-8}T+0.65972\times 10^{-11}T^{2}$$

## References

[1] Whitson C.H., Brulé M.R. (2000). Phase Behavior.

[2] Pedersen K.S., Christensen P.L., Shaikh J.A. (2014). Phase Behavior of Petroleum Reservoir Fluids.

[3] Won K.W. (1986). Thermodynamics for solid solution-liquid-vapor equilibria: Wax phase formation from heavy hydrocarbon mixtures. Fluid Phase Equilib..

[4] Pedersen K.S. (1995). Prediction of cloud point temperatures and amount of wax precipitation. SPE Prod. Facil..

[5] Lira-Galeana C., Firoozabadi A., Prausnitz J.M. (1996). Thermodynamics of wax precipitation in petroleum mixtures. AIChE J..

[6] Coutinho J.A. (1998). Predictive UNIQUAC: A new model for the description of multiphase solid-liquid equilibria in complex hydrocarbon mixtures. Ind. Eng. Chem. Res..

[7] Bishnoi P.R., Gupta A.K., Englezos P., Kalogerakis N. (1989). Multiphase equilibrium flash calculations for systems containing gas hydrates. Fluid Phase Equilib..

[8] Ballard A.L., Sloan E.D. (2004). The next generation of hydrate prediction: Part III. Gibbs energy minimization formalism. Fluid Phase Equilib..

[9] Segtovich I.S.V., Barreto A.G., Tavares F.W. (2016). Phase diagrams for hydrates beyond incipient condition-Complex behavior in methane/propane and carbon dioxide/iso-butane hydrates. Fluid Phase Equilib..

[10] Mahabadian M.A., Chapoy A., Burgass R., Tohidi B. (2016). Development of a multiphase flash in presence of hydrates: Experimental measurements and validation with the CPA equation of state. Fluid Phsase Equilib..

[11] Thomas F.B., Bennion D.B., Bennion D.W., Hunter B.E. (1992). Experimental and theoretical studies of solids precipitation from reservoir fluid. J. Can. Pet. Technol..

[12] MacMillan D.J., Tackett J.E., Jessee M.A., Monger-McClure T.G. (1995). A unified approach to asphaltene precipitation: Laboratory measurement and modeling. J. Pet. Technol..

[13] Nghiem L.X., Coombe D.A. (1997). Modelling asphaltene precipitation during primary depletion. SPE J..

[14] Almehaideb R.A. (2004). Asphaltene precipitation and deposition in the near wellbore region: A modeling approach. J. Pet. Sci. Eng..

[15] Orr F.M., Yu A.D., Lien C.L. (1981). Phase behavior of CO_2_ and crude oil in low-temperature reservoirs. Soc. Pet. Eng. J..

[16] Henry R.L., Metcalfe R.S. (1983). Multiple-phase generation during carbon dioxide flooding. Soc. Pet. Eng. J..

[17] Turek E.A., Metcalfe R.S., Fishback R.E. (1988). Phase behavior of several CO_2_/west Texas-Reservoir-Oil Systems. SPE Reserv. Eng..

[18] Michelsen M.L. (1982). The isothermal flash problem. Part I. Stability. Fluid Phase Equilib..

[19] Michelsen M.L. (1982). The isothermal flash problem. Part II. Phase-split calculation. Fluid Phase Equilib..

[20] Gupta A.K., Bishnoi P.R., Kalogerakis N. (1991). A method for the simultaneous phase equilibria and stability calculations for multiphase reacting and non-reacting systems. Fluid Phase Equilib..

[21] Mehra R.K., Heidemann R.A., Aziz K. (1982). Computation of multiphase equilibrium for compositional simulation. Soc. Pet. Eng. J..

[22] Nghiem L.X., Li Y.K. (1984). Computation of multiphase equilibrium phenomena with an equation of state. Fluid Phase Equilib..

[23] Risnes R. (1984). Equilibrium calculations for coexisting liquid phases. Soc. Pet. Eng. J..

[24] Li Z., Firoozabadi A. (2012). Initialization of phase fractions in Rachford–Rice equations for robust and efficient three-phase split calculation. Fluid Phase Equilib..

[25] Nelson P.A. (1987). Rapid phase determination in multiple-phase flash calculations. Comput. Chem. Eng..

[26] Leibovici C.F., Neoschil J. (1995). A solution of Rachford-Rice equations for multiphase systems. Fluid Phase Equilib..

[27] Michelsen M.L. (1994). Calculation of multiphase equilibrium. Comput. Chem. Eng..

[28] Leibovici C.F., Nichita D.V. (2008). A new look at multiphase Rachford–Rice equations for negative flashes. Fluid Phase Equilib..

[29] Okuno R., Johns R., Sepehrnoori K. (2010). A new algorithm for rachford-rice for multiphase compositional simulation. SPE J..

[30] Rachford H.H., Rice J.D. (1952). Procedure for use of electronic digital computers in calculating flash vaporization hydrocarbon equilibrium. J. Pet. Technol..

[31] Leibovici C., Neoschil J. (1992). A new look at the Rachford-Rice equation. Fluid Phase Equilib..

[32] Whitson C.H., Michelsen M.L. (1989). The negative flash. Fluid Phase Equilib..

[33] Brooks W.B., Gibbs G.B., McKetta J.J. (1951). Mutual solubilities of light hydrocarbonwater systems. Pet. Refin..

[34] Roland C.H., Smith D.E., Kaveler H.H. (1941). Equilibrium constants for a gas-distillate System. Oil Gas J..

[35] Bukacek R.F. (1955). Equilibrium Moisture Content of Natural Gases.

[36] Hibbard R.R., Schalla R.L. (1952). Solubility of Water in Hydrocarbons.

[37] Haugen K.B., Firoozabadi A., Sun L. (2011). Efficient and robust three-phase split computations. AIChE J..

[38] Nichita D.V., Gomez S., Luna E. (2002). Multiphase equilibria calculation by direct minimization of Gibbs free energy with a global optimization method. Comput. Chem. Eng..

[39] Robinson D.B., Kalra H., Rempis H. (1978). The Equilibrium Phase Properties of A Synthetic Sour Gas Mixture and a Simulated Natural Gas Mixture.

[40] Kohse B.F., Heidemann R.A. (1992). Tricritical lines and multiphase equilibria in quaternary mixtures. Fluid Phase Equilib..

[41] Erbar J.H., Jagota A.K., Muthswamy S., Moshfeghian M. (1980). Predicting Synthetic Gas and Natural Gas Thermodynamic Properties Using a Modified Soave-Redlich-Kwong Equation of State.

[42] Reshadi P., Nasrifar K., Moshfeghian M. (2011). Evaluating the phase equilibria of liquid water+ natural gas mixtures using cubic equations of state with asymmetric mixing rules. Fluid Phase Equilib..

[43] Sabet N., Gahrooei H.R.E. (2016). A new robust stability algorithm for three phase flash calculations in presence of water. J. Nat. Gas Sci. Eng..

[44] Prausnitz J.M., Lichtenthaler R.N., de Azevedo E.G. (1998). Molecular Thermodynamics of Fluid-Phase Equilibria.

[45] Rowe A.M., Chou J.C. (1970). Pressure-volume-temperature-concentration relation of aqueous sodium chloride solutions. J. Chem. Eng. Data.

[46] Li Y.K., Nghiem L.X. (1986). Phase equilibria of oil, gas and water/brine mixtures from a cubic equation of state and Henry’s law. Can. J. Chem. Eng..

[47] Smith J.M., Van Ness H.C. (1975). Introduction to Chemical Engineering Thermodynamics.

[48] Lyckman E.W., Eckert C.A., Prausnitz J.M. (1965). Generalized reference fugacities for phase equilibrium thermodynamics. Chem. Eng. Sci..

[49] Buck A.L. (1981). New equations for computing vapor pressure and enhancement factor. J. Appl. Meteorol..

[50] Poling B.E., Prausnitz J.M., O’Connell J.P. (2000). The Properties of Gases and Liquids.

